# Study on the Fast Transient Process of Primary Equipment Operation in UHV Fixed Series Capacitors Based on PEEC Method

**DOI:** 10.3390/s25154662

**Published:** 2025-07-27

**Authors:** Baojiang Tian, Kai Xu, Yingying Wang, Pei Guo, Chao Xiao, Wei Han, Yiran Dong, Jingang Wang

**Affiliations:** 1 State Grid Henan Electric Power Company, Zhengzhou 450052, China; tianbaoj@163.com; 2 State Grid Corporation of China, Beijing 100032, China; xukai_011@163.com; 3 Central China Branch of State Grid Corporation of China, Wuhan 430200, China; 15926317495@126.com; 4 Electric Power Research Institute of State Grid Henan Electric Power Company, Zhengzhou 450052, China; sngeet@163.com (C.X.); hanwei_0371@126.com (W.H.); 5 State Key Laboratory of Power Transmission Equipment Technology, School of Electrical Engineering, Chongqing University, Chongqing 400044, China; 202211021168t@stu.cqu.edu.cn (Y.D.); jingang@cqu.edu.cn (J.W.)

**Keywords:** UHV FSC, fast transient overvoltage, multi-conductor system, EMI, primary equipment operation

## Abstract

This manuscript proposes a fast transient simulation method based on PEEC to model overvoltage caused by spark gap and disconnecting switch operations in UHV series compensation (FSC). It proposes a simulation method based on the Partial Element Equivalent Circuit (PEEC) for modeling the fast transient processes associated with the operation of primary equipment in UHV FSC. Initially, a multi-conductor system model for both primary and secondary equipment on the cascade platform is developed. Then, the lumped components′ modeling of primary equipment and secondary equipment is added on the basis of multi-conductor model. Through simulation, the rapid transient overvoltage of primary equipment and the electromagnetic disturbance of the secondary system are analyzed. The simulation results provide insights into the distribution of fast transient overvoltage and the transient electromagnetic disturbance along the bus, from the low-voltage bus to the high-potential platform, under various primary equipment operating conditions. These findings provide a basis for theoretical analysis of the layout of sensor devices on platform and the design of electromagnetic shielding for interference-prone systems on platform.

## 1. Introduction

UHV FSC are highly effective in enhancing the transient stability, transmission capacity, and voltage profile of ultra-high-voltage transmission systems by compensating for the inductive reactance of the transmission line [1,2,3]. On the FSC platform, in addition to the primary system components responsible for the core compensation function, a variety of secondary system devices are deployed to perform measurement and protection tasks for the primary equipment. These secondary devices are critical to ensuring the safe and reliable operation of the complete series compensation system [4,5].

During the operation of series compensation devices, transmission line faults can trigger the activation of spark gaps and the operation of disconnectors, potentially leading to malfunctions or failures of protection devices. In severe cases, these issues may compromise the safe operation of the UHV FSC [6,7]. Therefore, it is necessary to perform simulation analyses of the fast transient processes induced by primary equipment operations, focusing on the overvoltage signals between the low-voltage busbar and the high-potential platform, as well as the electromagnetic interference (EMI) affecting the secondary equipment. Such analyses are essential to characterize the electromagnetic transient behavior and to provide insights for improving the system’s electromagnetic compatibility and operational reliability.

Previous studies on the fast transient processes of the UHV FSC under transmission line fault conditions have primarily focused on the energy absorption characteristics of metal-oxide varistors (MOVs), the discharge frequency of capacitor banks, and the associated damping effects. References [8,9] established equivalent mathematical models of the FSC under various operating conditions, such as transmission line faults, and investigated the electromagnetic transient behaviors of MOVs. References [10,11] studied the overvoltage issues induced by switching operations using system equivalence methods. Reference [12] conducted simulation analyses of overvoltage in UHV FSC under lightning surge conditions. However, in these studies, the platform of the FSC was modeled as a single node within the simulation circuit, which fails to capture the local electromagnetic transient interactions among conductors on the platform. Given the presence of complex multi-conductor systems between the primary and secondary equipment on the series compensation platform, the low-voltage busbar and the high-potential platform cannot be approximated as an equipotential system. Therefore, it is necessary to model the multi-conductor system on the platform to enable accurate simulation and analysis of its transient behaviors.

Modeling of the multi-conductor system on series compensation platforms has traditionally relied on multi-conductor transmission line (MTL) theory to simulate electromagnetic transient processes. However, this approach only considers coupling between conductors within the same segment [13,14,15]. Given the non-uniform and tightly coupled characteristics of the multi-conductor systems on series compensation platforms, MTL-based methods are inadequate for accurately capturing the coupling between conductors across different segments. The Partial Element Equivalent Circuit (PEEC) method discretizes complex three-dimensional conductors and calculates partial circuit parameters, representing electromagnetic field couplings between conductors in the form of equivalent circuits, which can be analyzed using circuit-based methods. Currently, the PEEC approach has been widely applied in fields such as electromagnetic compatibility, antenna modeling, and transient analysis [16,17,18]. Therefore, the PEEC methodology can be adopted to model the multi-conductor systems on series compensation platforms.

Aiming at the above problems, this manuscript proposes a PEEC-based simulation and analysis method for fast transient processes of primary equipment operation on UHV series compensation platforms. Firstly, PEEC discrete modeling is carried out for the multi-conductor system on the cascade patch platform, and a hybrid modeling method of the multi-conductor system and the platform aggregate components is investigated. Secondly, the fast transient process under spark gap triggering and disconnecting switch operation is simulated and analyzed, and the study of overvoltage between low-voltage busbar and high-potential platform and electromagnetic harassment of secondary system is completed.

## 2. Partial Elemental Equivalent Circuit Modeling of Multi-Conductor Systems

Partial Element Equivalent Circuit (PEEC) modeling is a numerical analysis method based on Maxwell’s equations, which transforms complex electromagnetic field problems into discretized circuit models for solution. For the UHV FSC involving multi-conductor systems, the PEEC method establishes capacitance, inductance, and resistance networks to accurately characterize parasitic effects among conductors and their electromagnetic coupling with the compensation device. This approach enables effective analysis of the system’s dynamic behavior, parasitic phenomena, and electromagnetic compatibility characteristics, thereby providing a theoretical foundation for optimizing device design and improving operational performance.

### 2.1. Electric Field Integral Equation

For a space conductor, the electric field integral equation can be derived from Maxwell’s system of equations by considering the effects of all electric field sources within the conductor as shown in (1).

(1) $${\vec{E}}_{0}(\vec{r},t)=\frac{\vec{J}(\vec{r},t)}{\sigma }+\frac{\partial \vec{A}(\vec{r},t)}{\partial t}+\nabla \Phi (\vec{r},t)$$

Here, ${\vec{E}}_{0}(\vec{r},t)$ represents the external electric field generated by external excitation sources within the multi-conductor system; $\vec{J}$ denotes the conductor current density; $\vec{A}(\vec{r},t)$ is the vector magnetic potential; $\Phi (\vec{r},t)$ is the scalar electric potential; σ represents the electrical conductivity of the conductor; and $\vec{r}$ is the position vector.

For a multi-conductor system composed of *H* conductors, the vector magnetic potential at position $\vec{r}$ can be expressed as follows:

(2) $$\vec{A}(\vec{r},t)=\sum_{h=1}^{H}\frac{\mu }{4\pi }\int_{v_{h}}\tilde{H}(\vec{r},\vec{r}\prime )\vec{J}(\vec{r}\prime ,t\prime )dv\prime$$

Here, $\vec{r}\prime$ is the position vector of the source point, and t′ represents the time delay between the source and the field point, which can be expressed as follows:

(3) $$t\prime =t-\frac{\left|\vec{r}-\vec{r}\prime \right|}{c}{\left(\varepsilon_{r}\mu_{r}\right)}^{\frac{1}{2}}$$

Here, *c* denotes the speed of light; $\varepsilon_{r}$ is the relative permittivity; and $\mu_{r}$ is the relative permeability. The expression for $\tilde{H}(\vec{r},\vec{r}\prime )$ is given as follows:

(4) $$\tilde{H}(\vec{r},\vec{r}\prime )=\frac{1}{\left|\vec{r}-\vec{r}\prime \right|}$$

The scalar electric potential can be expressed as follows:

(5) $$\Phi (\vec{r},t)=\sum_{h=1}^{H}\frac{1}{4\pi \varepsilon }\int_{v_{h}}\tilde{H}(\vec{r},\vec{r}\prime )q(\vec{r}\prime ,t\prime )dv\prime$$

By setting the external electric field ${\vec{E}}_{0}(\vec{r},t)$ to zero and substituting Equations (2) and (5) into Equation (1), the electric field integral equation can be obtained as shown in Equation (6):

(6) $$\frac{\vec{J}(\vec{r},t)}{\sigma }+\sum_{h=1}^{H}\frac{\partial }{\partial t}\left[\frac{\mu }{4\pi }\int_{v_{h}}\tilde{H}(\vec{r},\vec{r}\prime )\vec{J}(\vec{r}\prime ,t\prime )dv\prime \right]+\sum_{h=1}^{H}\nabla \left[\frac{1}{4\pi \varepsilon }\int_{v_{h}}\tilde{H}(\vec{r},\vec{r}\prime )q(\vec{r}\prime ,t\prime )dv\prime \right]=0$$

### 2.2. Cell-Based Discretization of Multi-Conductor Systems

**Figure 1:**
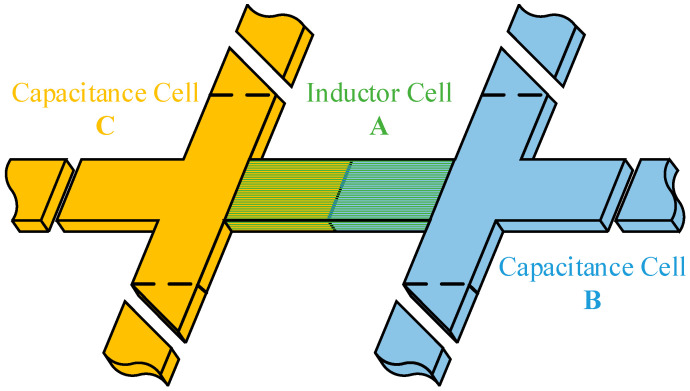
Discrete sectional methods for multi-conductor systems.

For the multi-conductor system on the FSC platform, a cell-based discretization must be performed prior to PEEC computation. After discretization, the system consists of inductive cells and capacitive cells. During the subdivision process, the length of each cell should be much smaller than the shortest wavelength of the electromagnetic waves considered in the simulation. Additionally, the aspect ratio of each cell should be moderate, and the boundaries of the capacitive cells should coincide with the midpoints of the adjacent inductive cells. The discretization method for the multi-conductor system is shown in Figure 1.

As shown in Figure 1, the multi-conductor system is discretized into one inductor cell, labeled A, and two capacitance cells, labeled B and C. The inductor cell A carries conduction current, while the capacitance cells B and C confine charges on their surfaces. Additionally, the boundaries of cells B and C coincide with the midpoints of the adjacent inductor cell.

### 2.3. Calculation of Partial Element Parameters

From the observation of Equation (6), it can be seen that the first term on the left-hand side represents the voltage drop caused by the conduction current along the direction of current flow. Assuming that the conductor has a cross-sectional area *a_i_* perpendicular to the current direction and a length *l_i_* along the current path, the voltage drop due to conduction current can be expressed as follows:

(7) $$V_{R}=\frac{l_{i}}{\sigma a_{i}}I$$

By analogy with the resistive voltage equation of V=RI, the equivalent partial resistance can be obtained as follows:

(8) $$R=\frac{l_{i}}{\sigma a_{i}}$$

The second term on the left-hand side of Equation (6) represents the voltage drop within the cell caused by its self-inductance and the mutual inductance with other cells:

(9) $$V_{L}=\sum_{j=1}^{H}\left[\frac{\mu }{4\pi }\frac{1}{a_{i}a_{j}}\int_{v_{i}}\int_{vj}\frac{{\vec{e}}_{i}\cdot {\vec{e}}_{j}}{\left|{\vec{r}}_{i}-{\vec{r}}_{j}\right|}dv_{j}dv_{i}\right]\frac{\partial I_{j}}{\partial t}=\sum_{j=1}^{H}Lp_{i,j}\frac{dI_{j}}{dt}$$

By analogy with the inductive voltage calculation formula of V=L⋅di/dt, the expression for the equivalent partial inductance can be obtained as follows:

(10) $$Lp_{i,j}=\frac{\mu }{4\pi }\frac{1}{a_{i}a_{j}}\int_{v_{i}}\int_{v_{j}}\frac{{\vec{e}}_{i}\cdot {\vec{e}}_{j}}{\left|{\vec{r}}_{i}-{\vec{r}}_{j}\right|}dv_{j}dv_{i}$$

Here, ${\vec{e}}_{i}$ and ${\vec{e}}_{j}$ are the unit vectors in the direction of the current densities on cells *i* and *j*, respectively, and *a_j_* is the cross-sectional area of cell *j*. When the cells are approximated as rectangular prisms, their axial current elements can be simplified as lying along the geometric centerlines. Under this approximation, the expression for the equivalent partial mutual inductance is simplified as follows:

(11) $$Lp_{i,j}=\frac{\mu }{4\pi }\int_{b_{j}}^{e_{j}}\int_{b_{i}}^{e_{i}}\frac{1}{\left|{\vec{r}}_{i}-{\vec{r}}_{j}\right|}d{\vec{l}}_{i}d{\vec{l}}_{j}$$

Here, the starting point and ending point of segment *i* are denoted as *b_i_* and *e_i_*, respectively, with a length of *l_i_*; and the starting point and ending point of segment *j* are denoted as *b_j_* and *e_j_*, respectively, with a length of *l_j_*. When segment *i* is perpendicular to segment *j*, the partial mutual inductance between them is zero.

**Figure 2:**
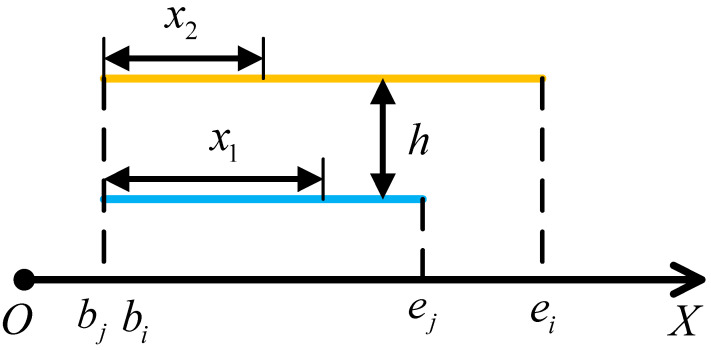
Schematic diagram of conductor parallel structure.

Considering that the multi-conductor system on the UHV series compensation platform is predominantly composed of mutually parallel structures, as shown in Figure 2, Equation (11) can be rewritten as follows:

(12) $$Lp_{i,j}=\frac{\mu }{4\pi }\int_{b_{j}}^{e_{j}}\int_{b_{i}}^{e_{i}}\frac{1}{\sqrt{{\left(x_{1}-x_{2}\right)}^{2}+h^{2}}}dx_{1}dx_{2}$$

Here, *x*_1_ and *x*_2_ are the lengths of the two segments along their common perpendicular line, and *h* is the parallel distance between the two segments.

By integrating Equation (12) and defining the indefinite integral *f* (*x*_1_,*x*_2_) as follows:

(13) $$f(x_{1},x_{2})=\frac{\mu }{4\pi }\left[\left(x_{1}-x_{2}\right)\ln(x_{2}-x_{1}+\sqrt{{\left(x_{2}-x_{1}\right)}^{2}+h^{2}})+\sqrt{{\left(x_{2}-x_{1}\right)}^{2}+h^{2}}\right]$$

The partial mutual inductance of a parallel-structured multi-conductor system can be expressed as follows:

(14) $$Lp_{i,j}=f(e_{j},e_{i})-f(e_{j},b_{i})-f(b_{j},e_{i})+f(b_{j},b_{i})$$

When *i* = *j*, the self-inductance of a rectangular conductor can be expressed as follows:

(15) $$Lp_{i,i}=\frac{\mu }{4\pi }\frac{1}{a_{i}^{2}}\int_{a_{i}}\int_{a_{i\prime }}\int_{0}^{l}\int_{0}^{l\prime }\frac{\left|d{\vec{I}}_{i}\cdot d{\vec{I}}_{i\prime }\right|}{r_{ii\prime }}da_{i}da_{i\prime }$$

When calculating the partial self-inductance of the multi-conductor system on the series compensation platform, the large conductor dimensions and irregular cross-sectional shapes make the use of Equation (15) computationally intensive. Therefore, for conductors with constant cross-sectional profiles under high-frequency conditions, the concept of Geometric Mean Distance (GMD) is introduced to simplify the calculation of partial self-inductance. The fundamental principle is to assume that the current is uniformly distributed along the perimeter of the conductor cross-section, and to replace the distance between two equidistant elemental lines with the GMD of the conductor’s perimeter [19]. The formula for calculating the geometric mean distance is given as follows:

(16) $$\ln m=\frac{1}{l^{2}}\int_{l}\int_{l}\ln\beta dl_{1}dl_{2}$$

Here, *m* is the geometric mean distance over the conductor cross-section perimeter, and β is the distance between two infinitesimal length elements *l*_1_ and *l*_2_ along the perimeter.

By replacing the distance between two elemental lines with the geometric mean distance, the partial self-inductance of a conductor with a constant cross-section can be expressed as follows:

(17) $$Lp_{i,i}=\frac{\mu l}{2\pi }(\ln\frac{2l}{m}-1)$$

The potential vector Φ of the capacitive cells can be expressed as follows:

(18) Φ=P×Q

Here, $\Phi \in R^{C}$ is the potential vector of the capacitive cells, $P\in R^{C\times C}$ is the partial potential coefficient matrix, and $Q\in R^{C}$ is the charge vector of the capacitive cells. The partial potential coefficients can be expressed as follows:

(19) $$p_{m,k}=\frac{1}{S_{m}S_{k}}\frac{1}{4\pi \varepsilon }\int_{S_{m}}\int_{S_{k}}\frac{1}{\left|\vec{r}-\vec{r}\prime \right|}ds_{k}ds_{m}$$

Here, $s_{m}$ and $s_{k}$ are the two surface cells corresponding to the mutual potential coefficient to be calculated, and $S_{m}$ and $S_{k}$ represent their respective areas.

## 3. Modeling of the UHV Series Compensation Platform

**Figure 3:**
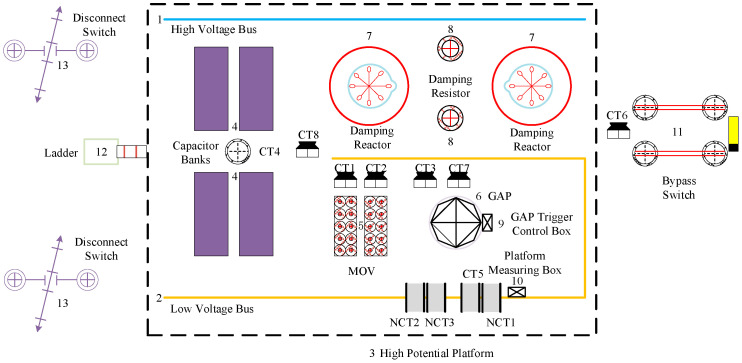
Layout of UHV FSC.

The UHV FSC primarily consists of primary equipment such as series capacitor banks, metal-oxide varistors (MOVs), damping circuits, high- and low-voltage busbars, spark gaps, disconnector, and bypass circuit breakers, as well as secondary equipment including energy acquisition current transformers (CTs), measurement CTs, and spark gap trigger control boxes. A schematic diagram of a typical UHV FSC layout is shown in Figure 3.

Among them, CT1 and CT2 are MOV current measurement CTs; CT3 is the platform flashover CT; CT4 is the capacitor bank unbalance protection CT; CT5 is the low-voltage busbar current measurement CT; CT6 is the bypass current measurement CT; CT7 is the spark gap current measurement CT; CT8 is the capacitor bank overcurrent protection CT; NCT1 is the platform CT for the measurement equipment box; NCT2 and NCT1 are the platform CTs of the measuring equipment box; NCT2 and NCT3 are the energy extraction CTs of the bypass GAP trigger control box.

### 3.1. Modeling of the Multi-Conductor System in the UHV FSC

**Figure 4:**
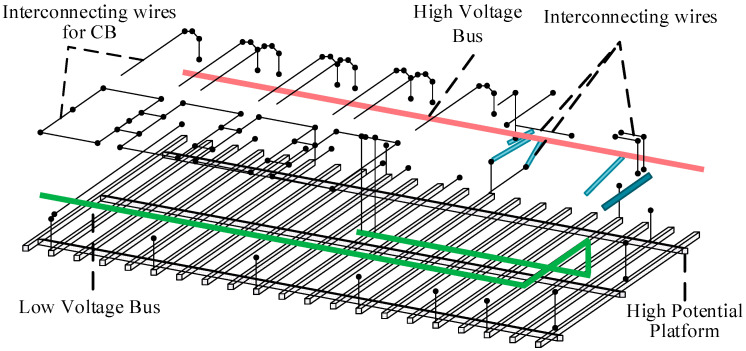
Multi-conductor system for the primary equipment of UHV FSC.

In addition to the high-potential platform and the primary equipment installed on it, the connecting conductors linking various pieces of primary equipment are also critical components that must be considered in the modeling of the UHV FSC [20]. The multi-conductor system formed by these connecting conductors, together with the high-potential platform and the high-voltage and low-voltage busbars, can accurately reflect the electromagnetic transient processes induced by primary equipment operations in the simulation. The multi-conductor modeling of the primary system on the UHV FSC platform is shown in Figure 4.

As shown in Figure 4, the high-potential platform is composed of a steel framework. Based on the installation locations of equipment such as MOVs, capacitor banks, and damping circuits, the primary multi-conductor system is discretized into segments. The connecting conductors are categorized into those linked to the capacitor banks and those connected to other primary equipment. The discrete unit length is set to Δ*l* = 100 mm. After discretizing the multi-conductor system into segments, the partial circuit parameters of each part are calculated using the previously described PEEC method.

Each discrete cell, through its geometric dimensions and material parameters, is equated to a set of series resistive (R) and inductive (L) elements, with a distributed ground capacitance to the ground, which is used to simulate its energy storage and discharge characteristics. For spatially neighboring conductor segments, mutual inductance and mutual capacitance are further calculated based on their spacing and relative positions to describe the inductive and capacitive coupling paths in the multiconductor system, and through the above process, the complex geometrical structure is systematically transformed into a sparse equivalent circuit network.

**Figure 5:**
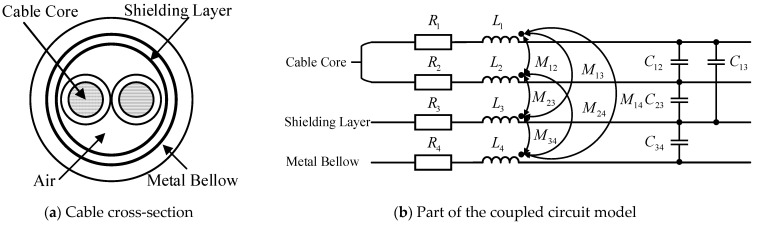
Multi-conductor system for the secondary equipment of UHV FSC.

In the UHV FSC, CT are connected to the secondary measurement boxes via shielded cables. The shielded cable and its outer metallic conduit together form a multi-conductor system [21]. The modeling of this multi-conductor system is illustrated in Figure 5.

Figure 5a shows the cross-sectional view of the multi-conductor system formed by the shielded cable and the corrugated metallic conduit. The shielded cable consists of two core wires, a shielding layer, and an outer corrugated metal conduit. By discretizing the core wires, shielding layer, and metal conduit into multiple segments and calculating the corresponding partial circuit parameters, the coupled multi-conductor circuit of a cable segment can be obtained, as shown in Figure 5b. In Figure 5b, *R*_1_–*R*_4_ represent the partial resistances of the core wires, shielding layer, and metal conduit; *L*_1_–*L*_4_ denote their partial self-inductances; *M_ij_* indicates the partial mutual inductances between different conductors; and *C_ij_* represents the partial mutual capacitances.

### 3.2. Modeling Method for Lumped Elements in the UHV FSC

In addition to the high-potential platform and the primary multi-conductor system, it is also necessary to establish models for the primary equipment installed on the platform. Devices such as capacitor banks and damping circuits can be modeled using lumped elements. However, the transient process triggered by the spark gap is more complex and can be represented by a structure consisting of a capacitor *Ca* in parallel with a time-varying resistor *Ra*. The calculation formula for the time-varying resistance is given as follows:

(20) $$R_{a}(t)=r_{a}+R_{0}e^{-t/\tau }$$

Here, $r_{a}$ is the steady-state conduction resistance of the spark gap, $R_{0}$ is the insulation resistance of the spark gap, and τ is the discharge time constant.

**Figure 6:**
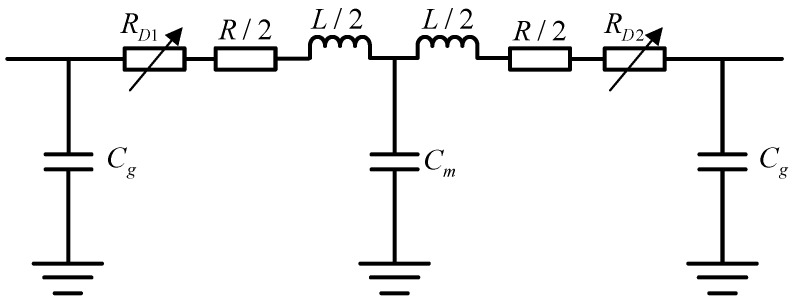
Equivalent circuit of series disconnector.

The series disconnector in the primary equipment adopts a two-port structure, and its equivalent circuit is shown in Figure 6.

The capacitances *C_g_* and *C_m_* represent the parasitic capacitance of the conductors at the two ends of the switch and the central conductor with respect to the ground, respectively, originating from the electrostatic coupling formed between the insulator support structure and the suspended conductor and the ground. For modeling, both are in the form of lumped parameters without considering the frequency characteristics and spatial distribution, and the capacitance value is set to 80 pF to reflect the influence of the structural layout on the transient characteristics of the system.

The fracture resistance *R*_*D*_ (including *R*_*D*1_, *R*_*D*2_) is used to simulate the leakage channel between conductors of the isolating switch in the disconnected state with weak arc conductance, and its physical sources include the air gap, residual ionization in the surface, and surface capacitance. The physical sources include the residual ionization in the air gap, the conductivity of humid contaminated surfaces and the residual conductivity during the arc extinguishing process. The modeling can be set to a fixed resistance value or a time-varying nonlinear function as required, which is used to reflect the non-ideal interruption behavior of the disconnectors during high-frequency transient processes.

**Figure 7:**
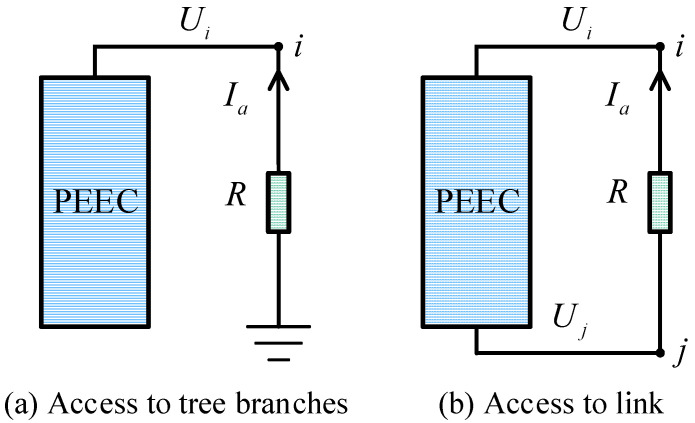
Topology schematic when the resistor is connected to the PEEC.

When lumped elements such as capacitors, resistors, and inductors are connected to a complex multi-conductor system, it is necessary to study the impact of these elements on transient calculations. Each added lumped element branch introduces a new state variable to the multi-conductor system, thereby requiring the addition of a new state equation. Taking the resistor as an example, Figure 7 shows its connection to the PEEC tree branches and link.

As shown in Figure 7, when the additional branch current *I*_a_ is selected as the state variable, the corresponding additional equation that needs to be introduced is as follows:

(21) $$\left\{\begin{array}{ll} U_{i}+R\times I_{a}=0 & \;\mathrm{to}\;\mathrm{tree}\;\mathrm{branches}\\ U_{i}-U_{j}+R\times I_{a}=0 & \;\mathrm{to}\;\mathrm{link} \end{array}\right.$$

Due to the unavailability of detailed electrical structures and circuit parameters for the MOVs installed on the high-potential platform from certain manufacturers, it is difficult to establish a broadband equivalent circuit model for the MOVs. In this study, a simplified model of the MOV is derived based on its voltage–current (V–I) characteristics. The V–I characteristic of the MOV can be expressed as follows:

(22) $$i=A{\left(\frac{u}{U_{n}}\right)}^{N}$$

Here, *A* is the constant of the V–I characteristic, *i* is the current flowing through the MOV, *u* is the voltage across the MOV, and $U_{n}$ is the reference voltage of the MOV. Based on the exponential derivation, the simplified model of the MOV can be expressed as follows:

(23) $$R=\frac{3}{6AT+AT^{3}u^{2}}$$

Here, *T* is the constant of the V–I characteristic. In this case, the MOV can be equivalently modeled by a voltage-controlled current source.

**Figure 8:**
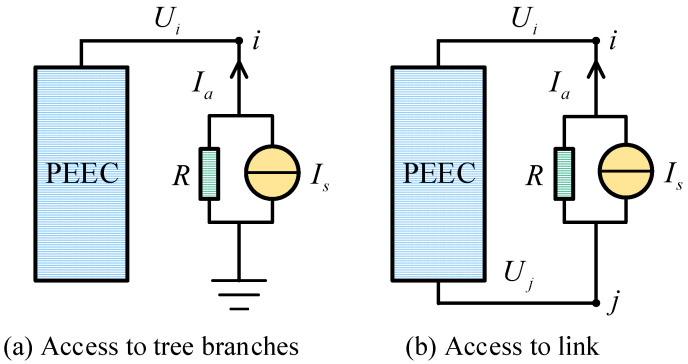
Topology schematic when current source is connected to PEEC.

When a current source is connected to a multi-conductor system, its internal resistance can be equivalently represented using a Norton circuit. The connection of the current source to the PEEC tree branches and link is shown in Figure 8.

By selecting the branch current *I*_a_ of the excitation source as the state variable, the corresponding additional equation can be expressed as follows:

(24) $$\left\{\begin{array}{ll} U_{i}+I_{a}\times R-I_{s}\times R=0 & \;\mathrm{to}\;\mathrm{tree}\;\mathrm{branches}\\ U_{j}-U_{i}-I_{a}\times R+I_{s}\times R=0 & \;\mathrm{to}\;\mathrm{link} \end{array}\right.$$

After modeling the primary equipment on the high-potential platform, it is also necessary to model the through-core CTs, the measurement box loads, and the loads within the trigger control boxes. Since the impedance characteristics of the through-core CTs and the loads vary with frequency, the broadband equivalent circuit modeling method proposed in [22] is adopted to establish the equivalent circuits for the CT impedance and the load impedance.

In order to ensure the effective capture of high-frequency components in the transient response process, this paper adopts a fixed time step Δ*t* = 0.2 ns, which satisfies the Nyquist sampling criterion. The sparse linear circuit network formed by PEEC modeling is transformed into a set of differential–algebraic equations in the time domain, and is discretized using the Backward Euler format, which is numerically stable and is especially. It has strong numerical stability and is particularly suitable for high steepness excitation scenarios such as lightning and electric arcs.

At each time step, the algebraic equations are solved iteratively by GMRES (generalized minimum residual method) with ILU (incomplete LU decomposition) preconditioner to accelerate the convergence, and the residual convergence threshold is set to 10^−6^. In order to avoid overshooting of the waveform at the early stage of the excitation, the waveforms are further discretized in the first 1 μs of the simulation, and the residual convergence threshold is set to 10^−6^. To avoid waveform overshoot in the early stage of excitation, the sampling density is further enhanced within 1 μs before simulation, and a damping adjustment term is introduced to improve the stability of the solution to ensure the numerical robustness and physical confidence of the model under the intense perturbation conditions.

## 4. Study on the Fast Transient Processes Induced by Series Compensation Device Operations

Based on the aforementioned modeling methods for the UHV FSC, a fast transient simulation model of the primary and secondary systems was established using the actual parameters of the 1000 kV UHV series compensation platform. The actual parameters of the UHV series compensation platform are listed in Table 1.

### 4.1. Simulation and Analysis of Fast Transient Processes in Primary Equipment

The fast transient processes triggered by spark gap activation and the closing of series disconnectors on the series compensation platform can induce high overvoltage between the low-voltage busbar and the platform. Additionally, the EMI coupled into the secondary system may result in measurement inaccuracies or even failures of secondary devices. Therefore, by first establishing a multi-conductor system model that includes the high-potential platform and busbars, and then connecting it with the equivalent circuit models of the primary equipment installed on the platform and the external disconnectors, the fast transient processes associated with primary equipment operations can be effectively simulated and analyzed [23].

Spark gaps are protective discharge devices in UHV FSC platforms and are installed on the parallel branch of the capacitor bank. The breakdown behavior of the spark gap is equated to a transient voltage source or a switched resistive element, which is used to simulate its transient disturbing excitation to the platform and secondary system. In modeling, it is usually regarded as a triggered excitation source or non-linear switch and is often used as the main excitation path in EMI analysis.

When simulating the overvoltage signals between the low-voltage busbar and the platform caused by spark gap triggering, the steady-state conduction resistance is set as *r_a_* = 0.5 Ω. The influence of different triggering voltages *U_b_* and discharge time constants τ on the overvoltage signals is considered. The grouping of different scenarios is shown in Table 2.

**Figure 9:**
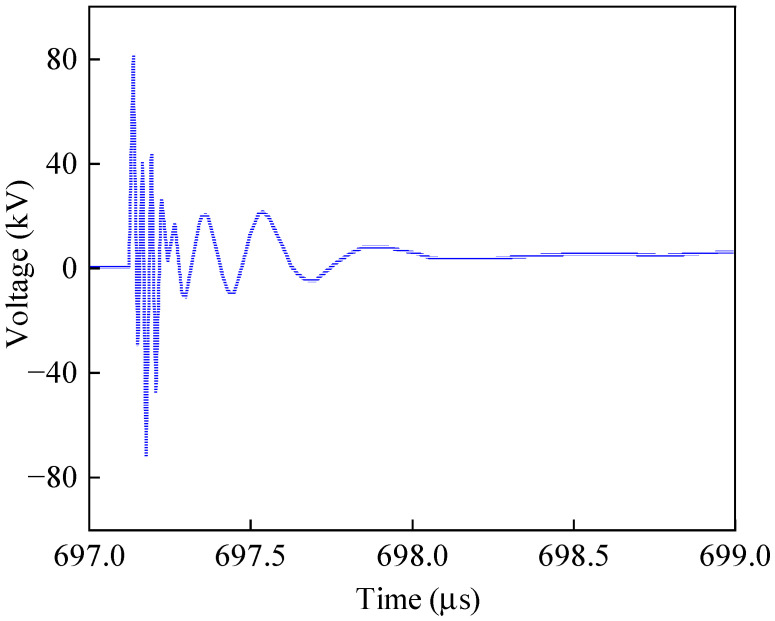
Spark gap trigger overvoltage time-domain waveform.

When the spark gap to trigger voltage *U_c_* = 320 kV, stable on-resistance *r_v_* = 0.5 Ω, discharge time constant τ = 1 ns by the conduction, G point and high-potential platform electromagnetic transient overvoltage waveforms between the platform, as shown in Figure 9.

**Figure 10:**
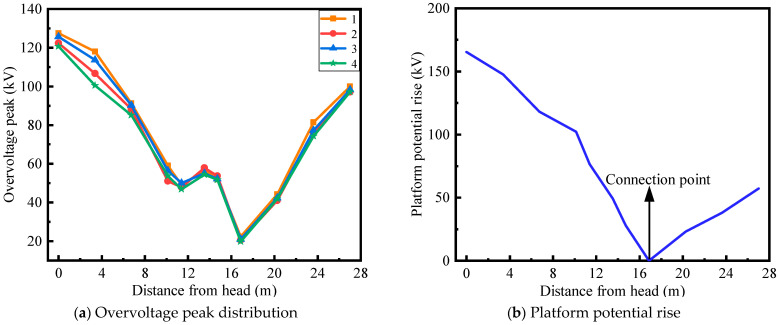
Spark gap trigger overvoltage.

The spatial distribution of peak voltage at each discrete node of the platform under spark gap breakdown excitation is shown in Figure 10. The peak value of each node is obtained by scanning its complete time-domain response (shown in Figure 9) and extracting the maximum value.

Neglecting the current flowing from the AC system into the UHV FSC, the distribution of the overvoltage peak values between the low-voltage busbar and the high-potential platform under different scenario groupings is shown in Figure 10a, and the platform potential rise is shown in Figure 10b.

As shown in Figure 10a, under different combinations of triggering voltages and discharge time constants, the overvoltage peak levels between the low-voltage busbar and the high-potential platform exhibit little variation as long as the spark gap is triggered. The lowest overvoltage peak appears at 16.25 m from the beginning of the busbar, which corresponds to the equipotential connection point between the low-voltage busbar and the high-potential platform. In contrast, the overvoltage levels at both ends of the busbar, farther from the equipotential point, are higher. As shown in Figure 10b, the overvoltage level is highest at the beginning of the busbar. This is because, at the instant of spark gap triggering, a strong transient current flows through the nearby region of the high-potential platform, resulting in a significant rise in the platform potential.

**Figure 11:**
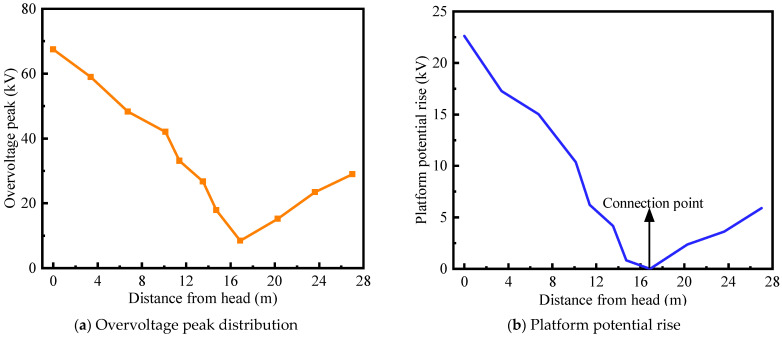
Isolated switch-off closing overvoltage.

Experimental analysis shows that the transient overvoltage generated during the closing of the disconnector is relatively high. Therefore, a simulation analysis of the fast transient process during disconnector closing is conducted. The distribution of the overvoltage and the platform potential rise during disconnector closing are shown in Figure 11.

As shown in Figure 11a, the fast transient overvoltage peak generated during disconnector closing is lower than that generated by spark gap triggering. This is because the transient current produced during disconnector closing is much smaller than that during spark gap triggering. Moreover, compared to spark gap triggering, the overvoltage peaks along the busbar decrease linearly from both ends toward the equipotential point. As shown in Figure 11b, the platform potential rise also decreases linearly from the busbar ends toward the equipotential point, and the magnitude of the platform potential rise is significantly lower than that observed in the spark gap triggering case.

From the above analysis, it can be concluded that the overvoltage levels induced by spark gap breakdown are higher than those caused by disconnector operations. For primary equipment installed far from the equipotential point, it is necessary to ensure that their transient withstood voltage levels exceed the overvoltage generated by spark gap triggering. For secondary devices and measurement systems, which are more susceptible to electromagnetic interference, it is recommended to install them at locations where the platform potential rise and the overvoltage levels between the low-voltage busbar and the platform are relatively low in order to prevent failures of the secondary equipment.

However, it should be noted that in the actual operating environment, fault triggering is somewhat random and unpredictable (e.g., lightning strikes, insulation degradation, partial discharges, etc.); so, it is not possible to accurately determine in advance the optimal location of the equipment corresponding to each fault. In addition, equipment installation is also constrained by various factors such as space, structure, cable routing and maintenance accessibility. Therefore, the optimal equipment placement proposal put forward in this paper should be understood as the interference distribution trend based on specific types of fault sources (e.g., spark gap breakdown) and typical wiring methods, and its applicability is mainly reflected in the following: guiding equipment placement to preferentially avoid coupling high-risk areas (e.g., close to spark gaps, platform edges), and proposing a quantitative reference method for layout design in electromagnetic environments.

In terms of limitations, the proposal is mainly applicable to the analysis of typical scenarios under the modeling of specific interference sources, and still needs to be combined with different operating conditions, other types of interference sources and historical fault data for more comprehensive adaptability assessment.

### 4.2. Simulation and Analysis of Electromagnetic Interference in the Secondary System

**Figure 12:**
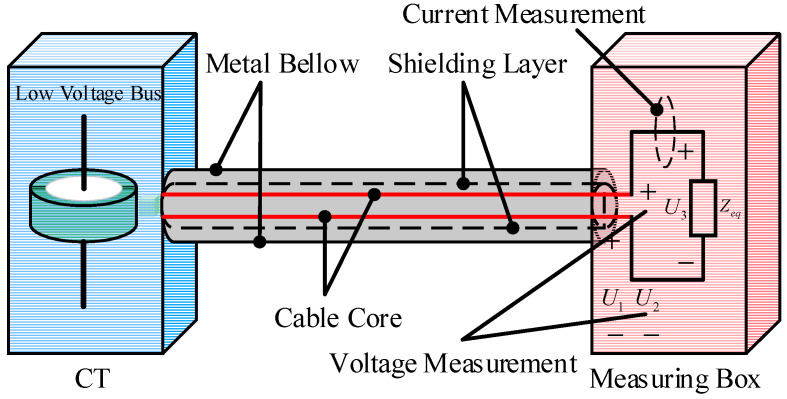
Secondary system connection.

In the UHV FSC, during spark gap triggering and disconnector operations, fast transient overvoltage is not only generated within the primary system but also coupled into the secondary system circuits through common impedance, mutual inductance, and capacitive coupling, resulting in EMI. The schematic diagram of the connection between the secondary measurement box and the energy acquisition CT is shown in Figure 12.

Based on the primary system model of the UHV series compensation platform, the secondary system circuit model—comprising the energy acquisition CT, shielded cable, and the equivalent load impedance of the measurement box—is incorporated. Subsequently, a simulation analysis is conducted to study the EMI induced in the secondary system during spark gap triggering and disconnector operations.

**Figure 13:**
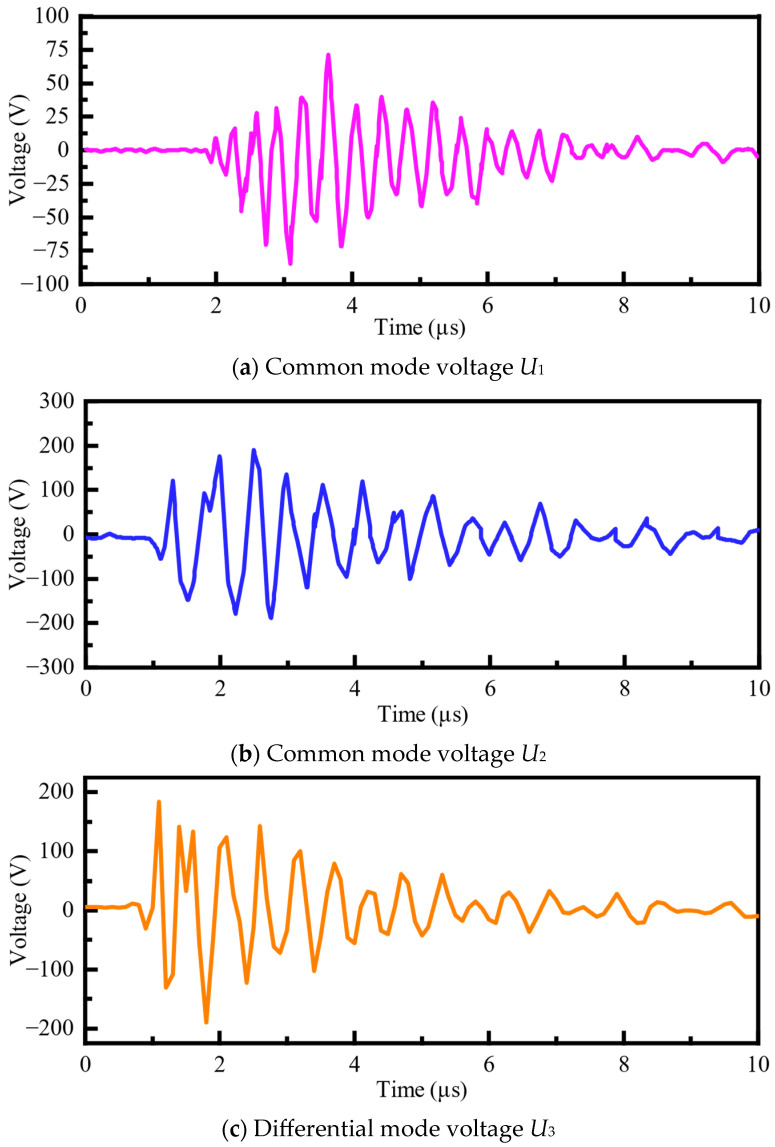
Typical disturbance voltage waveforms for secondary systems.

As shown in Figure 12, the simulation analyzes the common mode voltages *U*_1_ and *U*_2_, and the differential mode voltage *U*_3_ generated by spark gap triggering and disconnector operations. Typical disturbance voltage waveforms are presented in Figure 13.

As shown in Figure 13, the typical disturbance voltage waveforms in the secondary system exhibit durations on the order of several tens of microseconds, with the waveforms gradually decaying over time. Time–frequency analysis reveals that the disturbance signals contain a portion of high-frequency components along with a significant amount of low-frequency components, with the maximum frequency reaching several hundred megahertz. Energy spectral density analysis shows that the dominant frequencies and the majority of the signal energy are concentrated within the frequency band below 20 MHz, while the energy of the high-frequency components is relatively low and decays rapidly. Therefore, when analyzing the electromagnetic immunity of secondary equipment, appropriate suppression measures should be considered specifically for disturbances within this frequency range.

It should be noted that Figure 11 shows a schematic diagram of the connection between the platform components, which adopts a simplified wiring structure and idealized equivalent modeling method, and fails to completely include the structural details, material parameters, and terminal load characteristics of the actual cable. Therefore, the transient voltage waveforms in Figure 12 calculated based on this structure are mainly used to illustrate the typical response trend under spark gap excitation, rather than being universally adaptable to all engineering scenarios.

The transient EMI characteristics of the common-mode and differential-mode voltages under spark gap triggering and disconnector operation conditions are summarized in Table 3 and Table 4.

As shown in Table 3 and Table 4, the transient common-mode voltage peak-to-peak value induced by spark gap triggering can reach up to 1146 V, while the transient differential-mode voltage peak-to-peak value can reach 674 V. Time–frequency analysis of the disturbance voltages indicates that the dominant frequency components are concentrated below 5 MHz, suggesting a significant presence of low-frequency content. It can also be observed that the amplitude of the electromagnetic interference generated by spark gap triggering is greater than that generated by disconnector operations.

**Figure 14:**
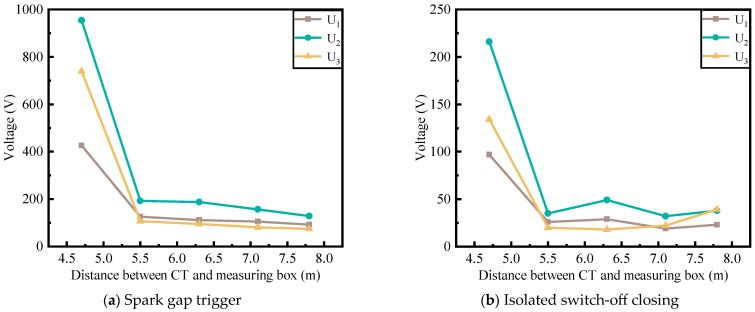
Peak disturbance voltage at different spacing.

Assuming that the energy acquisition CT and the measurement box are aligned along a straight line and the connecting shielded cable is laid out along this path, a simulation is conducted to analyze the disturbance voltages generated by spark gap triggering and disconnector operations under different distances between the CT and the measurement box. The variation in the peak disturbance voltage with respect to the distance is shown in Figure 14.

As shown in Figure 14, the peak values of disturbance voltages generated by spark gap triggering and disconnector operations decrease as the distance between the energy acquisition CT and the measurement box increases. This is because, at shorter distances, stronger electromagnetic interference is coupled into the measurement box load circuit via common impedance, inductive impedance, and capacitive impedance from the CT. Therefore, in the practical layout of the series compensation platform, it is advisable to increase the straight-line distance between the CT and the measurement box and to route the shielded cable along this straight path.

Since the CTs on the high-potential platform are located relatively close to the low-voltage busbar, a simulation analysis is conducted to evaluate the overvoltage signals between the CT enclosure and the high-potential platform under spark gap triggering and disconnector operation conditions. The simulation results are summarized in Table 5.

As shown in Table 5, the overvoltage peak generated between the CT enclosure and the busbar during spark gap triggering is higher than that generated during disconnector operation. Therefore, when designing the insulation of the CT enclosure, the overvoltage levels induced by spark gap triggering should be used as the reference.

This study is based on a PEEC-based transient simulation framework with the following assumptions:

(1) All conductors are considered ideal with uniform cross-sections and constant material properties (e.g., copper, σ = 5.8 × 10^7^ S/m);
(2) The spark gap is modeled as an idealized voltage-controlled switch, without accounting for arc channel dynamics or thermal effects;
(3) The dielectric medium is assumed to be homogeneous and linear (i.e., air), and effects such as corona, space charge accumulation, or humidity are neglected;
(4) The grounding network and platform support structures are simplified as ideal connections or lumped impedance elements.

Reference [19] discretized a typical 500 kV cascade platform into a complex multi-conductor system, as shown in Figure 4, and used the method in this paper to simulate the fast transient overvoltage in the cascade during gap breakdown. The results show that the overvoltage between capacitor node 64 on the LV busbar, which should be at equipotential, and the corresponding core-piercing current transformer (CT) case (equipotential to capacitor node 66 on the gap LV lead) exceeded 100 kV, which is much higher than the design withstand level of the CT’s insulating jacket for lightning overvoltage, and is in line with the flashover phenomenon in the project.

## 5. Conclusions

This manuscript proposes a PEEC-based method for studying the fast transient processes induced by primary equipment operations on the UHV FSC. By modeling the multi-conductor systems of both the primary and secondary equipment on the platform, simulations were conducted to analyze the fast transient overvoltage in the primary system and the electromagnetic interference experienced by the secondary system. Based on experimental results, the following conclusions are drawn.

(1) By modeling the multi-conductor systems of the primary and secondary equipment on the UHV FSC and connecting them with other lumped elements, it is possible to simulate the overvoltage distribution along the low-voltage busbar and the electromagnetic interference experienced by the secondary equipment under spark gap triggering and disconnector operation conditions. This approach addresses the issue of limited waveform recording data available under such transient events.

(2) The simulation results of fast transient overvoltage in the primary system indicate that, as long as the spark gap is triggered, the peak overvoltage between the low-voltage busbar and the high-potential platform remains relatively consistent under different triggering voltages and time constants. Under both spark gap triggering and disconnector operation conditions, the lowest overvoltage level occurs at the connection point between the low-voltage busbar and the high-potential platform, while the overvoltage level increases with distance from this equipotential point along the busbar. Moreover, the fast transient overvoltage induced by spark gap triggering is significantly higher than that caused by disconnector operation. Therefore, secondary equipment that is sensitive to electromagnetic interference should be installed near the equipotential connection point, whereas the transient withstood voltage level of the primary equipment located farther from this point should be higher than the overvoltage level generated by spark gap triggering.

(3) The simulation results of electromagnetic interference in the secondary system indicate that the disturbance voltage contains a significant number of low-frequency components, with the dominant frequency and energy concentrated below 20 MHz. The high-frequency components decay rapidly, suggesting that electromagnetic interference suppression measures should be primarily implemented within the frequency range below 20 MHz. In addition, the magnitude of electromagnetic interference caused by spark gap triggering is greater than that caused by disconnector operations. Therefore, the spark gap triggering condition should be used as a reference in the design of electromagnetic shielding for the measurement box and insulation for the CT enclosure. When arranging the positions of the energy acquisition CT and the measurement box, it is recommended to increase the straight-line distance between them and to route the shielded cable along this path.

## Figures and Tables

**Table 1:** Actual platform parameters.

Parameter	Value
Capacitor Banks	164.6 μF
Nominal Operating Voltage	98.4 kV
Minimum Spark Gap Triggering Voltage	1.8 p.u.
MOV Overvoltage Protection Level	2.3 p.u.
Platform Length	27 m
Platform Width	12 m
Platform Height from the Ground	11.87 m
Low Voltage Bus Height above Platform	1.5 m
Conductor Cross-section	30 mm × 5 mm
Conductor-to-Conductor Spacing	200 mm
PEEC Cell Length Δl	100 mm
Partial Self-inductance	1.3 μH/m(BUS)
Partial Mutual Capacitance	20~50 pF/m

**Table 2:** Different scenario groups.

Group	*U_b_*/kV	*τ*/ns
1	320	1
2	320	10
3	300	1
4	300	10

**Table 3:** Spark gap trigger EMI characterization.

	*U*_1_	*U*_2_	*U*_3_
Peak-to-Peak Value (V)	523	1146	674
Maximum Rise Time (μs)	5.1	4.3	6.8
Duration (μs)	6.5	11.2	10.5
Main Frequency (MHz)	0.3, 1.48, 2.6	0.5, 1.9, 2.6, 8.7	0.04, 1.32, 1.9

**Table 4:** Disconnecting switch operation EMI characterization.

	*U*_1_	*U*_2_	*U*_3_
Peak-to-Peak Value (V)	104	232	149
Maximum Rise Time (μs)	3.7	2.5	1.06
Duration (μs)	4.5	8.1	9.4
Main Frequency (MHz)	0.05, 0.4, 1.2	0.4, 1.2, 2.5	0.05, 0.4, 1.3

**Table 5:** Overvoltage between CT housing and low voltage bus.

	Spark Gap Trigger Overvoltage Peak (kV)	Isolated Switch-Off Closing Overvoltage Peak (kV)
Measuring box energy extraction CT	65.39	48.16
Trigger control box energy extraction CT	74.57	50.34

## Data Availability

The data used in the analysis presented in this paper will be made available, subject to the approval of the data owner.
